# Dietary Patterns and Cognitive Decline in Older Adults: Findings from the Western Australia Memory Study

**DOI:** 10.3390/nu18162592

**Published:** 2026-08-07

**Authors:** Carolina Blagojevic Castro, Samantha L. Gardener, Farzana Jahan, Juliana Chen, Belinda M. Brown, Ruey L. Loo, Kevin Taddei, Stephanie R. Rainey-Smith, Michael Weinborn, Ana Caroline R. dos Reis, Manohar L. Garg, Ralph N. Martins, Hamid R. Sohrabi

**Affiliations:** 1 Centre for Healthy Ageing, Health Futures Institute, Murdoch University, Murdoch, WA 6150, Australia; farzana.jahan@murdoch.edu.au (F.J.); belinda.brown@murdoch.edu.au (B.M.B.); stephanie.raineysmith@murdoch.edu.au (S.R.R.-S.); 2 Alzheimer’s Research Australia, Nedlands, WA 6009, Australia; samantha.gardener@uwa.edu.au (S.L.G.); kevin.taddei@uwa.edu.au (K.T.); ralph.martins@mq.edu.au (R.N.M.); 3 School of Psychology, Murdoch University, Murdoch, WA 6150, Australia; 4 Macquarie Medical School, Faculty of Medicine and Health Sciences, Macquarie University, Sydney, NSW 2109, Australia; manohar.garg@mq.edu.au; 5 The UWA Medical School, The University of Western Australia, Perth, WA 6009, Australia; 6 School of Medical and Health Sciences, Edith Cowan University, Joondalup, WA 6027, Australia; 7 Lifestyle Approaches Towards Cognitive Health Research Group, Murdoch University, Murdoch, WA 6150, Australia; 8 School of Mathematics, Statistics, Chemistry and Physics, College of Science, Technology, Engineering and Mathematics, Murdoch University, Murdoch, WA 6150, Australia; 9 Faculty of Medicine and Health, The University of Sydney, Camperdown, NSW 2006, Australia; juliana.chen@sydney.edu.au; 10 Australian National Phenome Centre and Centre for Computational and Systems Medicine, Health Futures Institute, Murdoch University, Perth, WA 6150, Australia; rueyleng.loo@murdoch.edu.au; 11 School of Psychological Science, The University of Western Australia, Crawley, WA 6009, Australia; michael.weinborn@uwa.edu.au; 12 Department of Life Sciences, State University of Bahia (UNEB), Salvador 41150-000, BA, Brazil; carolreis7295@gmail.com

**Keywords:** Alzheimer’s disease, *APOE* ε4 genotype, attention, cognition, cognitive decline, dietary patterns

## Abstract

Background: Preventive strategies, including adherence to specific dietary patterns, have received increasing attention as approaches to reduce cognitive decline and dementia risk. However, findings remain inconsistent, partly due to differences in dietary assessment methods, cognitive outcomes, and THE consideration of genetic susceptibility factors such as apolipoprotein E (*APOE*) genotype. Methods: This study examined associations between dietary patterns and longitudinal cognitive change in 185 older adults without dementia from the Western Australia Memory Study. Participants completed a food frequency questionnaire at baseline and underwent comprehensive neuropsychological assessments at baseline and up to five follow-up visits at 18-month intervals. Composite scores were generated for six cognitive domains and the Preclinical Alzheimer’s Cognitive Composite (PACC). Results: Western and Prudent dietary pattern scores were analysed using linear mixed-effects models stratified by *APOE* ε4 carrier status. Among *APOE* ε4 carriers, greater adherence to a Western dietary pattern was associated with a faster decline in attention (*p* = 0.016), whereas no significant associations were observed for the Prudent dietary pattern or among *APOE* ε4 non-carriers. This association was attenuated after excluding participants with possible baseline cognitive impairment (MoCA < 23). Conclusion: These findings suggest that adherence to a Western dietary pattern may accelerate cognitive decline in individuals genetically at risk for Alzheimer’s disease, highlighting the importance of considering gene–diet interactions when developing dietary strategies for dementia prevention.

## 1. Introduction

Enhanced healthcare and nutrition have significantly contributed to increased life expectancy worldwide. According to the World Health Organization [1], global life expectancy increased by over six years from 2000 to 2019, primarily due to improvements in medical care, sanitation, and nutrition. However, this increase in longevity has led to a higher prevalence of age-related conditions, notably Alzheimer’s disease (AD) [2]. Cognitive decline, characterized by the gradual deterioration of higher cortical functions such as memory, language, attention, and executive function, can progress to dementia, with AD being the most prevalent form [1,2,3].

While there is currently no cure for AD, early intervention and preventive strategies demonstrate great potential. One potential intervention strategy that is commonly accessible is adhering to specific dietary patterns, which encompass a combination of both nutrient and non-nutrient components [4].

Previous research on the association between dietary patterns and cognitive decline has produced varied results [5,6]. Townsend et al. [7] systematically reviewed 93 studies assessing the relationship between dietary patterns and cognitive outcomes. The results showed that 52% of prospective studies (of which 38 studies/41% of all prospective studies were cross-sectional in nature) and 50% of randomized controlled trials (RCTs) found that adherence to healthy dietary patterns was associated with slower cognitive decline and a reduced risk of cognitive disorders, such as dementias. Additionally, while healthy dietary patterns were frequently studied, fewer studies examined the impact of unhealthy dietary patterns, such as the Western diet or an “inflammatory diet,” with only 41% linking them to poorer cognitive outcomes.

Gardener et al. [5] observed that greater adherence to an Australian-style Mediterranean diet (AusMeDi) was inversely associated with executive function decline in apolipoprotein E (*APOE*) ε4 allele carriers, while higher adherence to an unhealthy Western diet was linked to a greater decline in visuospatial functioning in *APOE* ε4 allele non-carriers. No significant associations were found between the Prudent diet and cognitive performance. The findings suggest that the relationship between dietary patterns and cognitive function may be influenced by genetic factors, emphasising the need for genotype-specific analyses in aging research [8].

In addition to genetic risk factors, the mixed results are also likely to be partially due to methodological heterogeneity, including differences in dietary assessments, cognitive outcome measures, and population characteristics [7]. A common limitation of these studies is the relatively short intervention or follow-up periods (typically 2–3 years), which may not capture the long-term effects of dietary interventions on cognitive outcomes. This limitation highlights the need for additional longitudinal studies involving well-characterized aging populations, with a comprehensive assessment of various cognitive domains over time. In addition, many previous studies did not stratify analyses by *APOE* ε4 genotype despite evidence that genetic susceptibility may modify the relationship between diet and cognitive decline. Furthermore, several studies relied on global cognitive measures rather than domain-specific assessments, potentially overlooking subtle cognitive changes associated with dietary patterns. These methodological limitations may partly explain the inconsistent findings reported in the literature.

Our study aimed to explore the impact of two ‘a posteriori’ dietary patterns—the Western diet (an ‘unhealthy’ dietary pattern), and the Prudent diet (a ‘healthy’ dietary pattern)—on cognitive change over six timepoints (up to 90 months; average of 2.75 follow-up visits/50 months). We hypothesised that higher adherence to the Western dietary pattern is associated with greater cognitive decline, and conversely, higher adherence to the Prudent dietary pattern is associated with a slower rate of cognitive decline. The investigation was conducted on a well-defined cohort of older individuals drawn from the Western Australia Memory Study (WAMS) without significant cognitive impairment. To address previous methodological limitations, the present study applied a genotype-stratified approach based on *APOE* ε4 status, assessed changes across six cognitive domains using a comprehensive neuropsychological battery, and modeled cognitive trajectories over six waves spanning 90 months. These design features were intended to address several methodological gaps identified in previous studies, including limited genetic stratification, domain-specific cognitive assessments, and extended longitudinal follow-up.

## 2. Materials and Methods

### 2.1. Participants

This study was conducted using longitudinal data from 185 participants enrolled in the Western Australia Memory Study (WAMS), a large community-based cohort of older adults recruited between 2006 and 2009 to investigate molecular and neuropsychological predictors of cognitive decline. Participants were not excluded on the basis of baseline Montreal Cognitive Assessment (MoCA) score. A MoCA cut-off of <23 was subsequently applied in a sensitivity analysis to identify participants with possible baseline cognitive impairment. Participants recruited were community-dwelling older adults living in the metropolitan area of Perth, Western Australia. They were recruited through advertisements in various media, as well as via word of mouth, to participate as volunteers in a longitudinal research project. Baseline exclusion criteria included clinically uncontrolled psychiatric disorders (i.e., major depressive or anxiety disorders), a history of schizophrenia spectrum and other psychotic disorder, and a history of stroke or other neurological diseases (e.g., dementia any type, Parkinson’s disease), affecting cognition. Participants were included if, at baseline, they were able to (i) provide informed consent, (ii) complete neuropsychological battery; (iii) complete the Cancer Council of Victoria Food Frequency Questionnaire (CCVFFQ), and (iv) donate a blood sample for genotyping.

The WAMS received ethical approval from the Ramsay Health Care Human Research Ethics Committee (Reference No. 2003/ETH/0139, 7 October 2003). Written informed consent was obtained from all participants prior to participation in any study procedures. This study was reported according to the Strengthening the Reporting of Observational Studies in Epidemiology (STROBE) guidelines for cohort studies. The completed STROBE checklist is provided in Supplementary Table S4.

### 2.2. Cognitive Assessment

A comprehensive neuropsychological battery of well-validated measures was administered at baseline and at up to five additional timepoints, each 18 months apart (up to 90 months; average of 2.75 follow-up visits/50 months).

**Figure 1:**
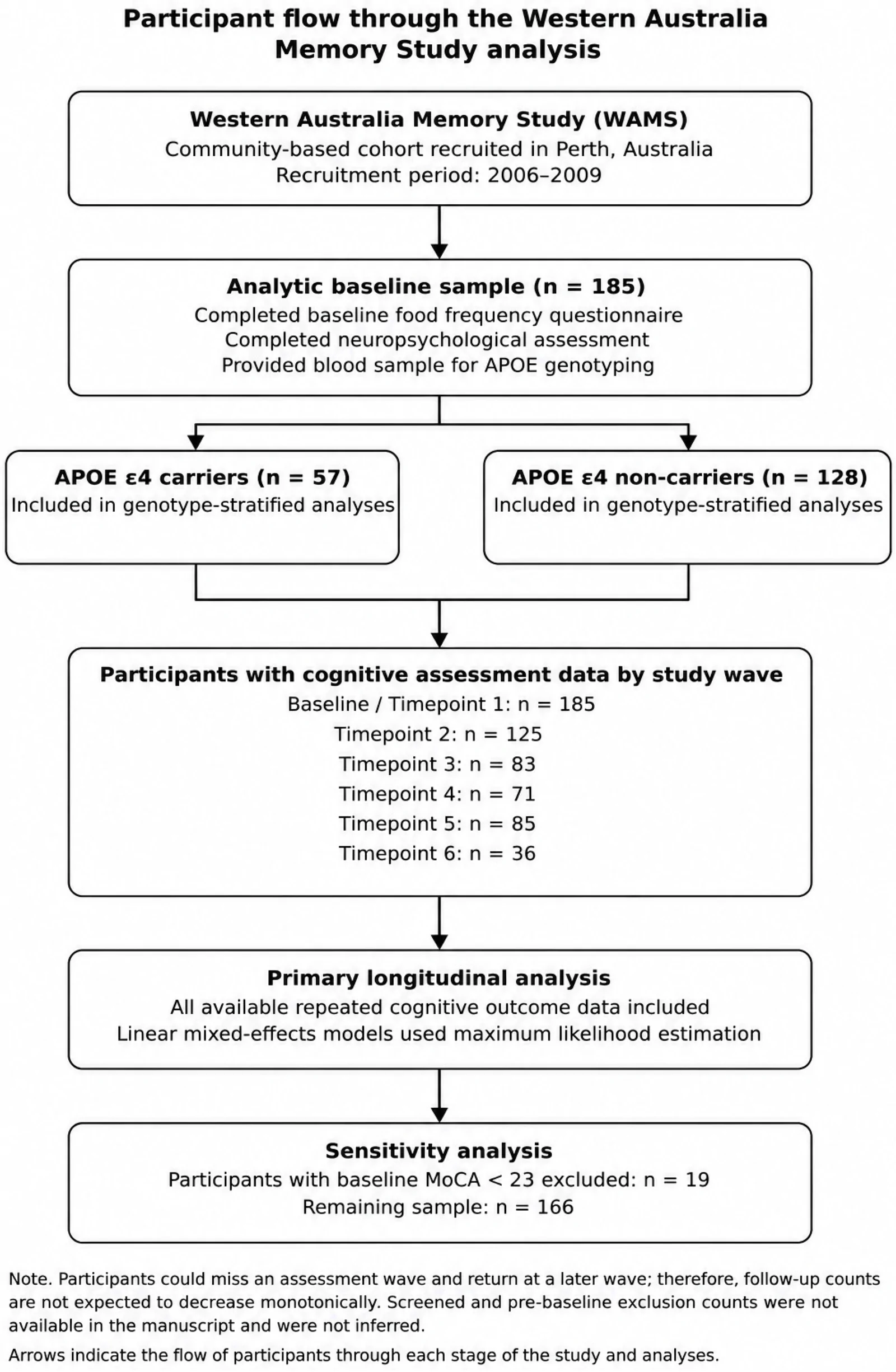
Participant flow diagram. Abbreviations: *APOE*: Apolipoprotein E, *APOE* ε4: Apolipoprotein E epsilon 4 allele, MoCA: Montreal Cognitive Assessment.

One hundred and twenty-five participants had second timepoint cognitive assessments, 83 participants had third timepoint cognitive assessments, 71 participants had fourth timepoint cognitive assessments, 85 participants had fifth timepoint cognitive assessments, and 36 participants had sixth timepoint cognitive assessments (Figure 1).

The battery assessed six cognitive domains (language, executive function, episodic memory, attention, recognition memory, and premorbid intelligence quotient (IQ)) in addition to global cognition. Composite scores were calculated for each of the six domains by converting raw scores to Z scores and averaging them to compute a single composite score for each domain. In this study, cognitive domain composites were constructed using an established theory- and test-based approach: raw scores from well-validated neuropsychological tests were Z-standardized and averaged to generate a single composite per domain (language, executive function, episodic memory, attention, recognition memory, premorbid IQ), and an adapted PACC (Preclinical Alzheimer’s Cognitive Composite) score adapted from prior models to reflect the cognitive tests available in the WAMS cohort (Table 1). For global cognition, MoCA score was converted to Z scores. As with the other composites, raw scores for tests included in the PACC were Z-standardized and averaged to form the PACC composite. Table 1 lists neuropsychological tests used to calculate domain-specific composite scores as well as the PACC.

### 2.3. Dietary Assessment

The CCVFFQ was completed at baseline to assess the consumption of 74 dietary questionnaire items, providing a comprehensive overview of participants’ usual dietary intake over the previous 12 months. During data processing, questionnaire items that contained multiple food or beverage components were disaggregated, resulting in 101 individual food and beverage items for analysis. Food composition data used to calculate daily nutrient intake were based on the Nutrient Tables for Use in Australia 1995 (NUTTAB95) [16]. Participants with extreme energy values were removed (<800 or >4000 kCal per day for males and <500 or >3500 kCal per day for females).

#### Western and Prudent Dietary Patterns

The 101 individual food and beverage items were subsequently classified into 33 predefined food groups to minimise within-person variation in the intake of individual foods. Principal component analysis (PCA) was then performed to derive dietary patterns from the dietary intake data of our Australian older adult cohort [17,18], ensuring contextual relevance rather than relying on a priori indices developed in other populations. Although derived empirically using PCA, the Prudent dietary pattern reflects dietary behaviours commonly recognized as healthy in the literature, characterized by higher intakes of vegetables, fruit, whole grains, and plant-based foods, while remaining distinct from a priori dietary indices. Food groups were entered as weight in grams per day. The factors were rotated by a varimax procedure resulting in non-correlated factors in order to facilitate factor interpretability. In determining number of factors to retain, components with an eigenvalue >1.25, Scree tests [18], and interpretability were considered. A cut-off of 0.30 was used to determine factor loadings included in each pattern, and two major patterns were extracted, labelled the Western pattern (explained 10.87% total variance) and Prudent pattern (explained 7.99% total variance). The Western dietary pattern reflects a processed, energy-dense diet with limited plant-based diversity; in contrast, the Prudent dietary pattern reflects a nutrient-rich, plant-focused diet (Table 2 shows factor loadings for these patterns). The dietary pattern score was constructed by summing intakes of the food groups weighted by factor loadings. Although minor cross-loadings were assessed, no food groups demonstrated primary loadings ≥ 0.30 on both factors. Therefore, each food group was assigned to a single pattern based on its highest loading.

### 2.4. Apolipoprotein E Genotyping

Fasting blood samples obtained using standard venipuncture of the antecubital vein were collected into Ethylenediaminetetraacetic acid (EDTA) tubes containing Prostaglandin E1 (Sapphire Biosciences, Redfern, Australia, 33·3 Nanogram/millilitre (ng/mL) to prevent platelet activation. Whole blood was centrifuged to separate leucocytes. Deoxyribonucleic acid (DNA) was isolated from the leucocytes using Qiagen Midiprep kits (QIAGEN, Hilden, Germany), and *APOE* genotyping was performed using established polymerase chain reaction amplification and restriction enzyme digest techniques [19]. Formal quality-control metrics (e.g., genotyping call rates, duplicate samples, or blinded repeat genotyping) were not available for this historical dataset.

### 2.5. Statistical Analysis

All statistical analyses were performed using R version 4.4.1 (R Core) [20] and the Statistical Package For The Social Sciences (SPSS, Chicago, IL, USA) version 30. To address multiple comparisons, false discovery rate (FDR) correction was applied across tests (excluding demographics) using the Benjamini–Hochberg procedure to adjust for multiple comparisons across cognitive domains. This method was chosen to maintain statistical power and reduce the risk of Type II errors, which can be inflated by more conservative corrections such as Bonferroni. Statistical significance was determined using FDR-adjusted *p*-values with a threshold of *p* < 0.05. Means, standard deviations and percentages are provided in Table 3 for the entire cohort as well as following stratification by *APOE* ε4 carriage status. Independent samples *t*-test and chi-square (χ^2^) analyses were conducted to evaluate group differences, as appropriate.

To assess the cohort for cognitive impairment, a cut-off score MoCA of <23 was utilised as a distinction between normal and mild cognitive impairment [15,21,22]. Independent sample *t*-tests and χ^2^ analyses evaluated group differences between those with <23 MoCA score and ≥23 MoCA score [22]. The MoCA < 23 cut-off was applied pragmatically to identify participants with possible cognitive impairments for sensitivity analyses and was not intended to represent a diagnostic threshold.

Linear regressions were fitted to analyse baseline cross-sectional associations between cognitive composites (dependent variable) and dietary pattern (independent variable). Models included age, sex, years of education (≤12 years, or >12 years), body mass index (BMI), energy intake and *APOE* genotype (absence of ε4 allele vs. presence of either one or two ε4 alleles) as independent variables.

A linear mixed-effect model was fitted to analyse associations between cognitive composites (dependent variable) and dietary pattern (independent variable). Models used a random intercept by ID (which considers the multiple observations by each individual for the longitudinal data) with as the same independent variables as in the linear regressions. All mixed-effects models were estimated using a 95% confidence level, and regression coefficients are reported alongside their standard errors to reflect the precision of estimates (Table 4, Table 5, Table 6 and Table 7). This analysis was repeated following the stratification of the cohort by *APOE* genotype. Time was treated as a continuous variable, measured in months since baseline, to estimate linear changes in cognitive outcomes across the follow-up period. For the longitudinal analyses, the reported regression coefficients (β) represent the interaction between baseline dietary pattern score and time (diet × time), reflecting the association between dietary pattern adherence and the rate of cognitive change over the follow-up period. Nonlinear terms (e.g., time^2^) were not included in order to maintain model simplicity and interpretability and avoid overfitting, particularly given the sample size and *APOE* ε4 stratification [23].

Participants contributed varying numbers of repeated outcome measurements (baseline to timepoint 6). Linear mixed-effects models were fitted using maximum likelihood estimation, which allows the inclusion of all available outcome data per participant and provides valid inference under the Missing At Random (MAR) assumption for the outcomes [24,25]. Thus, unequal follow-up times did not require imputation, and all observed responses contributed to model estimation.

For baseline covariates, a small proportion of values was missing. We examined the missingness mechanism and found evidence consistent with MAR. In this setting, we applied complete-case analysis (case wise deletion), whereby only individuals with complete covariate information were included in the model. Complete-case analysis yields unbiased estimates when missingness depends only on observed covariates included in the model (covariate-dependent missingness, a form of MAR) and not on the outcome [25,26]. Given the small proportion of missingness, this approach was considered appropriate.

## 3. Results

### 3.1. Demographics

This cohort comprised participants (*n* = 185) from the WAMS (31.9% male) without significant cognitive impairment with an average age of 70.6 ± 6.0 years. Participants had an average BMI of 26.6 ± 4.2 kg/m^2^, 30.8% were *APOE* ε4 allele carriers (i.e., ε2/4, ε3/4 or ε4/4) and over 25% had 12 or fewer years of education (Table 3). When the cohort was stratified by *APOE* status, the *APOE* ε4 allele carriers had a higher percentage of males (*p* = 0.047) and lower BMI (*p* = 0.035) than *APOE* ε4 allele non-carriers. There was no statistically significant difference in their dietary pattern adherence. There were no differences in baseline characteristics or diet scores between those that had the 6th cognitive timepoint (*n* = 36) and those that did not (*n* = 149). 

Nineteen individuals scored below the 23-point MoCA cut-off for defining mild cognitive impairment. Baseline comparisons between groups (<23 MoCA score and ≥23 MoCA score) revealed no significant differences in energy intake, age, BMI, Western diet score, Prudent diet score, or *APOE* carriage. However, there were significant differences in the proportion of males (*p* = 0.041) and years of education (*p* = 0.005), with the <23 MoCA scoring group having a higher percentage of males (52.6% vs. 29.5%) and being more likely to have ≤12 years of education (52.6% vs. 22.9%).

Twenty-two (12%) of the cohort developed one or more cardiometabolic conditions over 90 months. Baseline comparisons between groups (those who developed cardiometabolic conditions and those who did not) revealed no significant differences in energy intake, sex, age, BMI, Prudent diet score, or *APOE* carriage (Supplementary Table S1). However, there were significant differences in the years of education (*p* = 0.006) and Western diet score (*p* = 0.013), with those who developed cardiometabolic conditions being more likely to have ≤12 years of education (50.0% vs. 22.7%), and a higher Western diet score (256.5 ± 195.3 vs. 185.9 ± 112.2).

### 3.2. Cross-Sectional Linear Regression Analyses

In cross-sectional linear regression analyses, following FDR adjustment, no significant associations were observed between dietary patterns and cognitive performance in the cohort as a whole (Table 4). Similarly, no significant associations were identified between dietary patterns and any cognitive domain among either *APOE* ε4 allele non-carriers or carriers after correction for multiple comparisons (Table 5).

### 3.3. Longitudinal Linear Mixed Models

**Figure 2:**
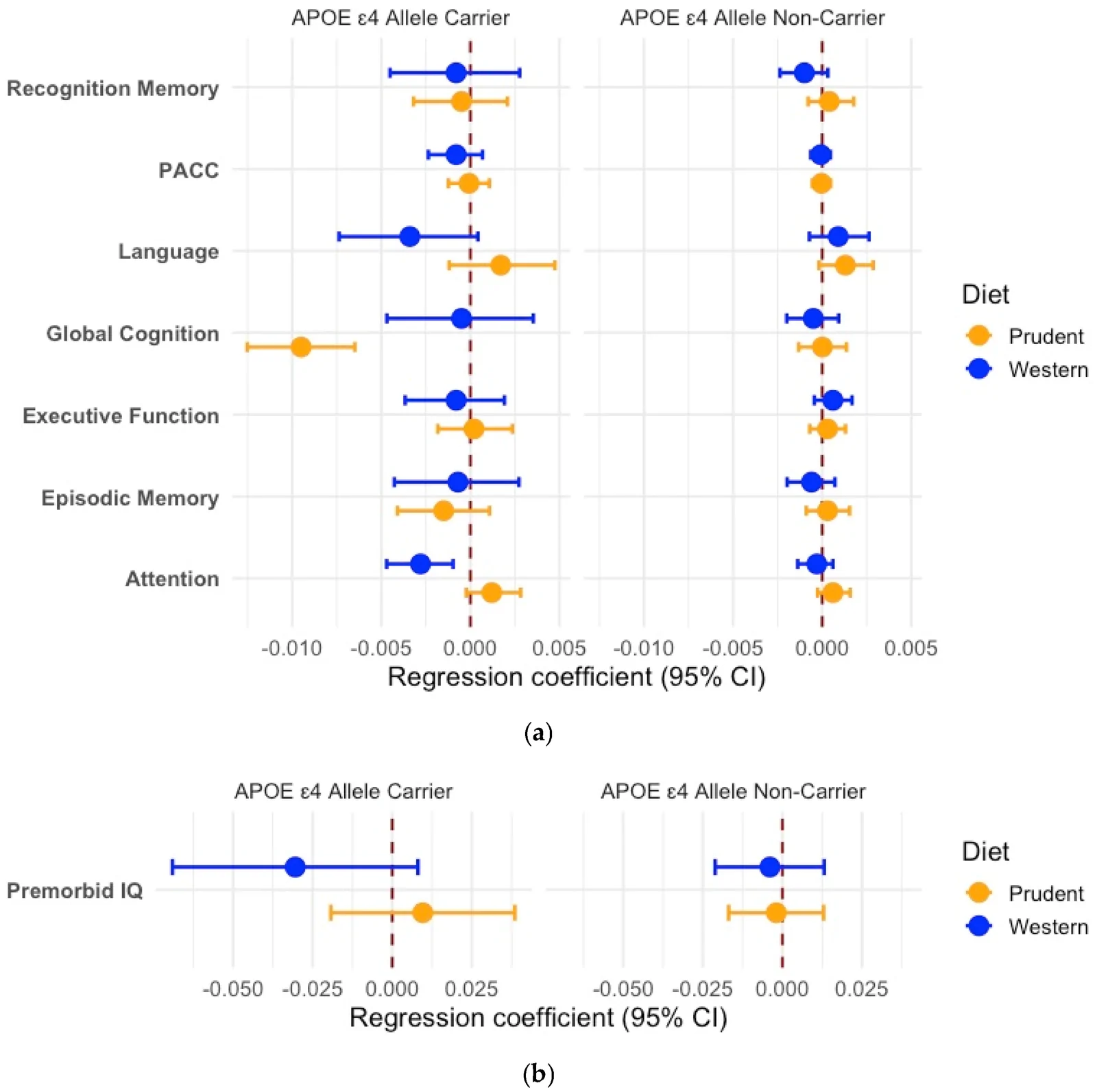
Regression coefficients (β) with 95% confidence interval of the effects of Western and Prudent diets on cognitive outcomes. (**a**) All cognitive outcomes except Premorbid IQ. (**b**) Premorbid IQ.

In linear mixed-effect models, there were no significant associations observed between dietary patterns and cognitive decline in the cohort as a whole (Table 6). Following FDR adjustment, Western diet score was negatively associated with the attention composite (β = −0.0028; SE = 0.00095; FDR-adjusted *p* = 0.016) in *APOE* ε4 allele carriers, such that higher Western diet adherence at baseline was associated with faster decline in the attention domain (Table 7, Figure 2). Partial eta squared for the significant effect between Western diet score and the attention composite is 0.18 (values over 0.14 typically interpreted as ‘large’ effect), indicating that a substantial proportion of variance is explained by the Western diet score [27].

There were no significant associations observed between dietary patterns and cognitive decline in *APOE* ε4 allele non-carriers (Table 7, Figure 2). This is to mention that the genotype-stratified longitudinal analyses were underpowered—particularly at the final wave due to attrition—necessitating cautious, exploratory interpretation of both significant and null findings. The null results for the Prudent diet should be interpreted with caution. Although our findings are consistent with prior studies reporting weak or absent associations, limitations in statistical power and variance must also be considered. Our retrospective design analysis [28] indicated a Type-S error probability of 0.047 on average, meaning that if an effect had been statistically significant, there was only a 4.7% chance of obtaining the wrong direction. However, the Type-M error was 3.41 the average, suggesting that any significant result would, on average, have overstated the true magnitude by more than threefold. This pattern indicates that, while the direction of the association can be estimated with some confidence, the study lacked precision to detect and accurately quantify modest dietary effects. Accordingly, the null finding for the Prudent diet is best understood as inconclusive rather than definitive, underscoring the importance of larger, adequately powered studies to clarify the role of Prudent dietary patterns.

In addition, to address potential reverse causality due to prodromal cognitive symptoms, we conducted a sensitivity analysis excluding participants with Montreal Cognitive Assessment (MoCA) scores < 23 at baseline (*n* = 19), which may indicate early cognitive impairment (Supplementary Tables S2 and S3). In our original analysis including all participants, higher Western diet adherence was significantly associated with a faster decline in attention among *APOE* ε4 carriers (β = −0.0028; SE = 0.00095; FDR-adjusted *p* = 0.016). However, after excluding participants with MoCA scores < 23, this association was attenuated and was no longer statistically significant (β = −0.0017; SE = 0.00092; FDR-adjusted *p* = 0.233; Supplementary Table S3).

Regression coefficients (β) and 95% confidence intervals for the association between Western and Prudent dietary pattern scores and cognitive composite outcomes were stratified by *APOE* ε4 carrier status. Estimates are derived from linear mixed-effects models adjusted for age, sex, education, energy intake and body mass index. Statistical significance was determined based on model-based inference with false discovery rate correction rather than visual inspection of confidence intervals. To enhance the visibility of the estimates and confidence intervals, Premorbid IQ is plotted separately (Figure 2b) because its markedly wider confidence interval compresses the scale and reduces the visibility of the other estimates in Figure 2a.

## 4. Discussion

### 4.1. Western Dietary Patterns and Cognitive Decline

This study examined the impact of two ‘a posterior’ dietary patterns—the Western diet (an ‘unhealthy’ dietary pattern) and the Prudent diet (a ‘healthy’ dietary pattern)—on cognitive change over six timepoints (up to 90 months). We observed a single significant longitudinal association after correction for multiple comparisons, whereby higher baseline Western diet adherence was associated with faster attention decline among *APOE* ε4 carriers. This finding should be interpreted cautiously, as no other cognitive domains remained significant after FDR correction, and the association was attenuated in the sensitivity analysis excluding participants with possible baseline cognitive impairment. There were no associations observed between dietary patterns and cognitive decline in the cohort as a whole or *APOE* ε4 allele non-carriers.

Our findings highlight the negative impact of consumption of the Western diet on specific cognitive function over time. In agreement with our findings, the Western diet, characterized by a high consumption of processed foods, sugars, and unhealthy fats, has previously been linked to negative outcomes, including cognitive decline and higher risk of dementia, although further intervention studies are needed to establish causality [29].

The mechanisms through which the Western diet negatively impacts cognition are believed to involve increased inflammation, oxidative stress, and insulin resistance [29]. These factors can lead to neurodegeneration and cognitive impairment. The prevailing understanding is that the unhealthy components of the Western diet may disrupt normal brain function and promote conditions that are detrimental to cognitive health [29,30]. Thus, the dietary choices made within this pattern could significantly influence cognitive aging and the risk of developing dementia [29,30]. The inflammatory and pro-oxidative properties of the Western diet may contribute to its negative impact on cognition, as certain components of this diet such as red meat, processed meats, sugars and refined grains can trigger neuroinflammation, a process associated with conditions including AD [29,30]. Our significant results were observed in the attention cognitive composite domain, and specific areas central to the control of spatial attention, such as the prefrontal cortex (PFC) [31], have been found to be particularly susceptible to the negative influence of the Western diet [5,30,32].

Studies highlight that attention, especially selective and sustained attention, can be disrupted even before impairment in language and visuospatial abilities become noticeable [33,34]. The Western diet, characterized by a high intake of saturated fats and refined sugars, has been linked to neuronal dysfunction and the impairment of prefrontal-dependent tasks, potentially via neuroinflammation, insulin resistance, and oxidative stress [35,36]. Specifically, diets rich in saturated fats can lead to region-specific metabolic dysfunction, with the PFC showing heightened vulnerability in early stages of diet-induced brain changes [37]. In addition, insulin resistance—a hallmark of poor dietary habits—has been associated with reduced functional connectivity and glucose metabolism in the PFC, contributing to deficits in attentional processes [38]. Furthermore, the effects of a Western diet are region-specific in the brain. For instance, alterations in neurotransmitter and synaptic plasticity markers have been observed in the frontal cortex, a region critical for attention and executive functions. These alterations suggest that the Western diet may specifically disrupt the neural circuits involved in attention [30,32].

Additionally, in the present cross-sectional analyses, no significant associations were observed between Western dietary pattern adherence and recognition memory in either *APOE* ε4 non-carriers or carriers following FDR correction. Although prior literature has suggested that recognition memory is particularly sensitive to hippocampal dysfunction, including disruptions in synaptic plasticity and insulin signalling, the current findings do not provide evidence that Western diet adherence is independently associated with performance in this cognitive domain at baseline. Nevertheless, experimental and observational studies have consistently implicated high intakes of saturated fats, refined carbohydrates, and pro-inflammatory foods in promoting hippocampal vulnerability through mechanisms such as neuroinflammation, impaired insulin signalling, and altered synaptic integrity [8,35].

The absence of statistically significant associations in the overall cohort and within *APOE* ε4-stratified analyses may reflect the complex interplay between genetic susceptibility and lifestyle factors. While Western-style dietary patterns have been linked to adverse metabolic and neuroinflammatory processes that can compromise hippocampal function, such effects may be subtle or require longer exposure to manifest in measurable cognitive deficits, particularly in relatively healthy or cognitively unimpaired populations [8,39]. Furthermore, in *APOE* ε4 carriers, the strong genetic predisposition to amyloid accumulation, neuroinflammation, and cerebral metabolic dysfunction may overshadow or attenuate detectable associations with modifiable lifestyle factors in cross-sectional analyses [40,41].

Taken together, these findings do not support a significant cross-sectional association between Western dietary patterns and recognition memory performance after correction for multiple comparisons. However, the biological plausibility of diet-related effects mediated through neuroinflammatory and insulin-resistance pathways remains well supported by prior research. In the absence of biomarker data, these mechanistic considerations should be interpreted as hypothesis-generating rather than explanatory, underscoring the need for longitudinal and biomarker-informed studies to clarify whether dietary exposures interact with *APOE* ε4 status to influence cognitive trajectories over time.

### 4.2. Prudent Dietary Patterns and Cognitive Outcomes

Our Prudent dietary pattern showed no association with cognitive decline, aligning partly with previous research reporting limited and mixed results [5,42]. Prudent dietary patterns are often excluded from systematic reviews due to their limited use and construction method. Chen et al. [43] reviewed two studies: the Australian Imaging, Biomarkers and Lifestyle (AIBL) study found no significant association with the Prudent dietary pattern and cognitive decline [5], while the Swedish National study on Aging and Care-Kungsholmen (SNAC-K) study reported a lower decline on the Mini-Mental State Examination with higher adherence [42].

Our study employed an ‘a posteriori’ dietary pattern analysis, which statistically derives dietary behaviours from real-world consumption patterns rather than predefined criteria. Unlike a priori methods, which classify diets as healthy or unhealthy, ‘a posteriori’ approaches capture the complexity of dietary habits influenced by cultural, social, and economic factors, allowing for a more nuanced understanding of diet–health relationships [44,45]. These data-driven patterns provide valuable insights for developing targeted dietary recommendations and interventions. While the identified patterns are reflective of common dietary habits in an Australian aging population, we acknowledge that dietary habits are influenced by cultural, social, and economic factors. Therefore, the Western and Prudent patterns identified in this study may not generalize to other populations with distinct dietary traditions. Future research should explore whether similar associations are observed in diverse cohorts to strengthen the external validity of these findings.

### 4.3. APOE Genotype and Dietary Interactions

We found a significant association between higher Western diet adherence at baseline and faster decline in attention in those at higher risk of AD due to carriage of an *APOE* ε4 allele. Such association was not observed in the *APOE* ε4 allele non-carriers. A study led by Samuelsson et al. [46] indicates that a Western-style dietary pattern is linked to changes in cerebrospinal fluid (CSF) biomarkers associated with preclinical AD. The study found that the associations between the Western diet and CSF biomarkers were more pronounced in *APOE* ε4 allele carriers compared to non-carriers. The findings reported by Samuelsson et al. [46] may indicate that the Western diet can exacerbate the underlying biological vulnerabilities present in *APOE* ε4 allele carriers, leading to an impact on cognitive function. Another possible explanation for the association reported on data from *APOE* ε4 allele carriers could be that the Western diet contributes to inflammation and oxidative stress, which are known to affect brain health. Therefore, *APOE* ε4 allele carriers may have a heightened inflammatory response, making them more susceptible to the negative effects of Western dietary patterns. In contrast, *APOE* ε4 allele non-carriers may have a different metabolic response to dietary patterns, potentially providing them with a degree of resilience against cognitive decline associated with a Western diet. While statistically significant, the observed effect sizes were small, and their clinical implications remain uncertain. This finding highlights the exploratory nature of the associations, which require replication in larger, diverse cohorts.

### 4.4. Differences Across MoCA Groups

Importantly, the significant association between Western diet adherence and attentional decline observed in *APOE* ε4 carriers did not persist after excluding participants with potential cognitive impairment (MoCA < 23), highlighting the fragility of this finding and supporting its interpretation as exploratory rather than conclusive.

Additionally, our study observed statistically significant sex differences between MoCA groups (scoring <23 and ≥23; *p* = 0.041), with a higher proportion of males in the <23 group (52.6% vs. 29.5%). This finding aligns with prior evidence suggesting demographic variations in cognitive screening outcomes, highlighting the potential role of sex-specific factors in cognitive performance and test sensitivity [47]. Future studies could further explore these differences to clarify underlying mechanisms. The significant difference in education levels between MoCA groups (scoring <23 and ≥23; *p* = 0.005) showed that participants in the <23 group were more likely to have 12 or fewer years of education (52.6% vs. 22.9%). This supports the well-documented association between education and cognitive performance [48]. While this finding strengthens the validity of the observed MoCA distinctions, it also suggests that education may act as a confounding variable, warranting cautious interpretation of group comparisons. These findings are limited by the observed differences in sex distribution and education levels between MoCA groups, which may influence the generalizability of the results. Future studies should consider stratifying analyses or adjusting for these variables, as was done in the current analysis, to better isolate the effects of other factors on cognitive outcomes.

### 4.5. Cardiometabolic Conditions and Cognitive Decline

In this study, 22 participants (12%) developed one or more cardiometabolic conditions over the 90 months of follow up. Analysis revealed that those who developed cardiometabolic conditions had an ‘unhealthier’ diet at baseline with higher adherence to the Western diet and reported lower education years, with a higher percentage having 12 or fewer years of education. Given the potential influence of cardiometabolic conditions on cognitive decline, additional analyses incorporating these conditions as confounders was conducted. However, the inclusion of this variable did not alter the primary findings. As the results remained unchanged, these additional analyses were not reported in detail but are noted in Supplementary Table S1. Future studies may also focus on participants with and without cardiometabolic conditions to examine their effects in relation to the Western diet on cognition.

### 4.6. Strengths and Limitations

The study capitalises on the well-defined dataset from the Western Australian Memory Study; however, the analysis is not without limitations. The CCVFFQ used in this study relied on participants’ self-reported estimates of their food intake over the past year. This method is commonly used in dietary studies but is known to have limitations, as it can lead to inaccuracies and misclassification of dietary adherence. This concern is particularly significant in studies involving cognitively impaired participants. To minimize this issue, we only included participants who were without significant cognitive impairment at the study’s outset when diet data was collected. While an objective measure that indicates dietary adherence, such as a nutrient biomarker panel, would ideally complement self-reported data, this method also has drawbacks, such as the inability to cover all possible nutrients. Relying on the baseline FFQ only assumes no change in dietary intake over the study period, which may overlook variations in participants’ eating habits, potentially influencing the results. However, previous research showed that the Western dietary pattern exhibited robust long-term stability over a three-year period [49]. To address this limitation, future studies could incorporate repeated dietary assessments throughout the follow-up period, enabling the capture of potential changes in dietary patterns over time. Additionally, the dietary data were analysed using the NUTTAB95 food composition database, as implemented by the Cancer Council of Victoria for the CCVFFQ. This may limit the nutritional accuracy of certain food components compared to more recent databases, potentially affecting the precision of dietary exposure estimates.

A key limitation of our study is the potential for reverse causality, particularly in participants with prodromal cognitive symptoms. Although formal clinical diagnoses were not available, we used MoCA scores to screen for potential impairment. Sensitivity analyses excluding participants with MoCA < 23 attenuated the observed association between Western diet and attentional decline in *APOE* ε4 carriers, suggesting that early cognitive changes may have influenced dietary habits or reporting accuracy. Consequently, in individuals without cognitive impairment, the influence of diet on cognitive trajectories may be more limited. Future studies should incorporate the clinical diagnosis of cognitive status and repeated dietary assessments to better clarify temporal relationships between diet and cognition.

Nonetheless, there are several strengths in our study that enhance the credibility of the results. The internal validity of our findings is improved with the majority of participants being Caucasian. Additionally, we adopted a rigorous approach by controlling for a wide range of demographic variables and using the FDR to adjust *p*-values, ensuring the robustness of our statistical outcomes. Furthermore, our findings are consistent with previous reported studies which describe a ‘healthy’ or ‘Prudent’ pattern in opposition to a ‘Western’ or ‘unhealthy’ dietary pattern [29].

Due to the stratification of analyses by *APOE* ε4 status, subgroup sample sizes were reduced, increasing the risk of both type I (false-positive) and type II (false-negative) errors. Therefore, findings from these stratified analyses—particularly those related to the association between Western diet and attention decline in *APOE* ε4 carriers—should be considered exploratory. Although *APOE* genotyping was conducted using established laboratory protocols, formal quality control metrics such as call rates, duplicate genotyping, or blinded repeat reads were not recorded for this dataset. The absence of these QC measures represents a methodological limitation and may affect the reliability of *APOE* classification. Accordingly, findings stratified by *APOE* ε4 status should be interpreted with caution. For future research, randomized double-blinded clinical trials should be conducted to investigate the impact of Prudent and Western diet interventions on composite cognitive scores. Additionally, such studies should aim to include larger, more diverse populations and consider long-term dietary adherence to better generalise the findings across different demographic groups and to assess the sustainability of cognitive benefits over time.

## 5. Conclusions

The current study suggests that, in an Australian older adult population, consuming components in an unhealthy dietary pattern may be associated with a greater decline in attention, specifically among *APOE* ε4 allele carriers. However, this association was attenuated in the sensitivity analysis excluding participants with possible baseline cognitive impairment, indicating that the findings should be interpreted with caution. This contrasts with the consumption of a healthy dietary pattern, which did not show a significant impact on cognitive function in our cohort.

Overall, these results highlight a potential interaction between dietary patterns and genetic susceptibility in cognitive aging. Given the observational nature of this study and the small effect sizes observed, the findings should be considered exploratory, and replication in larger, well-characterized longitudinal cohorts and intervention studies is warranted before firm clinical or public health recommendations can be made.

## Figures and Tables

**Table 1:** Summary of composite score structure.

Cognitive Domain	Neuropsychological Test	Outcome Measure	Reference
Premorbid IQ	Cambridge Contextual Reading Test	Total number of words correctly spoken	[9]
Language	Controlled Oral Word Association Task	Number of words produced in 1 min	[10]
Executive Function	D-KEFS verbal fluency Category Switching Category Fluency (Animals)	Number of words produced in 1 min	[10]
	Trail Making Test Part B	Time to complete	[11]
Episodic Memory	RCFT 30 min delay	Number of elements correctly recalled	[12]
	CVLT-II delayed recall	Number of words correctly recalled	[13]
Attention	Trail Making Test Part A	Time to complete	[11]
	Digit Span Total	Total correct trials (forwards & backwards)	[14]
Recognition Memory	CVLT-II d′	Discrimination index for recognition of words	[13]
	RCFT recognition hits	Number of elements correctly identified	[12]
Global Cognition	Montreal Cognitive Assessment	Total number of items correctly answered	[15]
PACC	Montreal Cognitive Assessment	Total number of items correctly answered	[15]
	RCFT 30 min delay	Number of elements correctly recalled	[12]
	Trail Making Test Part B	Time to complete	[11]
	COWA total	Number of words produced in 1 min	[10]
	Digit Span Total	Total correct trials (forwards & backwards)	[14]
	CVLT-II long delay free recall	Number of words correctly recalled	[13]

Abbreviations: CVLT-II, California Verbal Learning Test-II; d′, d prime; D-KEFS, Delis–Kaplan Executive Function System; IQ, intelligence quotient; PACC, Preclinical Alzheimer’s Cognitive Composite; RCFT, Rey Complex Figure Test.

**Table 2:** Factor loadings for the two major factors (diet patterns) identified from principal components analysis.

	Western Diet	Prudent Diet
Red meats	0.727	
Processed meats	0.614	
Chips	0.566	
Sweets	0.536	
Poultry	0.517	
Refined grains	0.512	
Condiments	0.449	
Potatoes	0.401	
Beer	0.391	
Other breakfast cereals	0.358	
Fish	0.335	
Meat pies	0.333	
Pizza	0.333	
Fruit juice	0.301	
Other vegetables		0.749
Dark-yellow vegetables		0.669
Green leafy vegetables		0.531
Fruit		0.505
Cruciferous vegetables		0.461
Garlic		0.405
Whole grains		0.361

**Table 3:** Descriptive statistics for the cohort who completed the CCVFFQ and subgroups of the cohort based on stratification by *APOE* ε4 allele carriage. If not otherwise described, data are presented as mean ± standard deviation of the mean.

	Total Sample (*n* = 185)	*APOE* ε4 Allele Carriers (*n* = 57)	*APOE* ε4 Allele Non-Carriers (*n* = 128)	*p*-Values for *APOE* ε4 Allele Carrier Status Differences
Age, y	70.6 ± 6.0	69.6 ± 4.6	70.8 ± 6.2	0.100
Sex, men; n (%)	59 (31.9)	24 (42.1)	35 (27.3)	**0.047**
Presence of *APOE* ε4 allele; n (%)	57 (30.8)			
Baselinebody mass index ^†^, kg/m^2^	26.6 ± 4.2	25.9 ± 3.3	27.2 ± 4.6	**0.035**
Baseline energy intake, Kcal	1590.6 ± 532.2	1617.6 ± 493.7	1599.2 ± 558.6	0.411
Education ≤ 12 y; n (%)	48 (25.9)	15 (26.3)	33 (25.8)	0.939
Baseline Western diet score	190.8 ± 126.4	195.3 ± 129.57	195.1 ± 125.3	0.445
Baseline Prudent diet score	220.5 ± 97.9	223.8 ± 85.4	221.8 ± 103.9	0.449

Characteristics compared using independent samples *t*-test for continuous variables and χ^2^ for categorical variables. ^†^ Body Mass Index is calculated as weight in kilograms divided by height in meters squared. Abbreviations: *APOE*, apolipoprotein E; Kcal, kilocalorie; Kg, kilogram; m^2^, meter squared; y, years. Bold indicates statistical significance (*p* < 0.05).

**Table 4:** Results of linear regression analyses examining the association between baseline diet scores and baseline cognitive performance. Standardised β values shown.

	Western Diet					Prudent Diet				
	β	SE	*p*-Value	FDR-Adjusted *p*-Value	95% CI	β	SE	*p*-Value	FDR-Adjusted *p*-Value	95% CI
**Premorbid IQ**	0.0032	0.0082	0.698	0.956	−0.0129, 0.0192	−0.0014	0.0068	0.837	0.890	−0.01468, 0.01189
**Language**	0.0002	0.0008	0.834	0.840	−0.0014, 0.0017	0.0015	0.0007	0.029	0.223	0.00016, 0.00291
**Executive Function**	0.0003	0.0005	0.613	0.817	−0.0008, 0.0013	0.0004	0.0005	0.390	0.540	−0.00053, 0.00138
**Episodic Memory**	−0.0007	0.0006	0.314	0.503	−0.0019– 0.0006	−0.0001	0.0006	0.866	0.866	−0.00123, 0.00103
**Attention**	−0.0002	0.0004	0.576	0.659	−0.0011, 0.0006	0.0005	0.0004	0.205	0.514	−0.00026, 0.00124
**Recognition Memory**	−0.001	0.0006	0.115	0.308	−0.0022, 0.0002	0.0004	0.0006	0.524	0.645	−0.00077, 0.00153
**PACC**	−0.0003	0.0004	0.432	0.692	−0.001, 0.0004	0.0003	0.0003	0.315	0.420	−0.00031, 0.00098
**Global Cognition**	−0.0008	0.0006	0.222	0.356	−0.002, 0.0005	0.0005	0.0006	0.938	0.938	−0.00111, 0.00121

Abbreviations: *APOE*, apolipoprotein E; CI, confidence interval; FDR, false discovery rate; IQ, intelligence quotient; PACC, Preclinical Alzheimer’s Cognitive Composite; SE, standard error. Model includes age, sex, years of education (≤12 years or >12 years), body mass index, energy intake and *APOE* genotype as independent variables.

**Table 5:** Results of linear regression analyses examining the association between baseline diet scores and baseline cognitive performance stratified by *APOE* ε4 allele carrier status. Standardised β values shown.

		Western Diet					Prudent Diet				
	*APOE* ε4 Allele Carrier Status	β	SE	*p*-Value	FDR-Adjusted *p*-Value	95% CI	β	SE	*p*-Value	FDR-Adjusted *p*-Value	95% CI
Premorbid IQ	+	0.001	0.026	0.950	0.980	−0.04995, 0.05323	0.004	0.024	0.864	0.996	−0.04458, 0.05316
Language	+	−0.003	0.002	0.124	0.616	−0.00780, 0.00086	0.001	0.001	0.254	0.779	−0.00134, 0.00523
Executive Function	+	−0.001	0.001	0.478	0.660	−0.00416, 0.00193	0.0005	0.001	0.666	0.777	−0.00178, 0.00280
Episodic Memory	+	−0.001	0.001	0.479	0.671	−0.00459, 0.0214	−0.0008	0.001	0.491	0.688	−0.00341, 0.00162
Attention	+	−0.001	0.009	0.066	0.232	−0.00355, 0.00006	0.0009	0.0007	0.195	0.684	−0.00046, 0.00236
Recognition Memory	+	−0.001	0.001	0.465	0.965	−0.00499, 0.00226	−0.0005	0.001	0.706	0.984	−0.00315, 0.00213
PACC	+	−0.001	0.001	0.155	0.758	−0.00448, 0.00064	0.001	0.001	0.193	0.851	−0.00068, 0.00364
Global Cognition	+	−0.0007	0.003	0.835	0.994	−0.00729, 0.00064	−0.0004	0.002	0.856	0.930	−0.00571, 0.00473
Premorbid IQ	-	0.008	0.0101	0.389	0.454	−0.01108, 0.28516	−0.010	0.008	0.221	0.351	−0.02844, 0.00651
Language	-	0.0007	0.0008	0.356	0.824	−0.00088, 0.00246	0.001	0.0007	0.059	0.208	−0.00004, 0.00298
Executive Function	-	0.0003	0.0005	0.536	0.751	−0.00079, 0.00153	0.0003	0.0005	0.544	0.762	−0.00074, 0.00142
Episodic Memory	-	−0.0007	0.0007	0.302	0.697	−0.00214, 0.00065	0.0003	0.0006	0.569	0.797	−0.00089, 0.00163
Attention	-	−0.0002	0.0005	0.633	0.799	−0.00124, 0.00075	0.0004	0.0004	0.352	0.821	−0.00481, 0.00135
Recognition Memory	-	−0.001	0.0007	0.088	0.206	−0.00258, 0.00016	0.0006	0.0006	0.361	0.505	−0.00070, 0.00193
PACC	-	−0.0001	0.0004	0.717	0.779	−0.00106, 0.00072	0.0002	0.0004	0.594	0.694	−0.00069, 0.00121
Global Cognition	-	−0.0008	0.0008	0.299	0.523	−0.00254, 0.00077	−0.0002	0.0008	0.814	0.891	−0.00197, 0.00154

Abbreviations: *APOE*, apolipoprotein E; CI, confidence interval; FDR, false discovery rate; IQ, intelligence quotient; PACC, Preclinical Alzheimer’s Cognitive Composite; SE, standard error. Model includes age, sex, years of education.

**Table 6:** Results of linear mixed-effect model analyses examining the association between baseline diet scores and change in cognitive performance over 90 months. Standardised β values shown.

	Western Diet					Prudent Diet				
	β	SE	*p*-Value	FDR-Adjusted *p*-Value	95% CI	β	SE	*p*-Value	FDR-Adjusted *p*-Value	95% CI
**Premorbid IQ**	−0.0060	0.0074	0.416	0.555	−0.02063, 0.00853	0.00001	0.0064	0.998	0.998	−0.01254, 0.01256
**Language**	0.0002	0.0007	0.721	0.721	−0.00123, 0.00177	0.00137	0.0006	0.045	0.183	0.00003, 0.00271
**Executive Function**	0.0004	0.0004	0.422	0.562	−0.00057, 0.00137	0.00037	0.0004	0.406	0.646	−0.00051, 0.00127
**Episodic Memory**	−0.0005	0.0006	0.401	0.642	−0.00179, 0.00071	−0.00025	0.0005	0.656	0.874	−0.00137, 0.00086
**Attention**	−0.0006	0.0004	0.123	0.329	−0.00150, 0.00017	0.00065	0.0003	0.094	0.379	−0.00010, 0.00141
**Recognition Memory**	−0.0008	0.0006	0.159	0.318	−0.00212, 0.00034	0.00030	0.0005	0.601	0.687	−0.00083, 0.00144
**PACC**	−0.0001	0.0002	0.543	0.621	−0.00069, 0.00036	0.00001	0.0002	0.961	0.961	−0.00046, 0.00048
**Global Cognition**	−0.0003	0.0007	0.603	0.690	−0.00175, 0.00102	−0.00041	0.0006	0.522	0.597	−0.00167, 0.00084

Abbreviations: *APOE*, apolipoprotein E; CI, confidence interval; FDR, false discovery rate; IQ, intelligence quotient; PACC, Preclinical Alzheimer’s Cognitive Composite; SE, standard error. Model includes age, sex, years of education (≤12 years or >12 years), body mass index, energy intake and *APOE* genotype as independent variables.

**Table 7:** Results of linear mixed-effect model analyses examining the association between baseline diet scores and change in cognitive performance over 90 months stratified by *APOE* ε4 allele carrier status. Standardised β values shown.

		Western Diet					Prudent Diet				
	*APOE* ε4 Allele Carrier Status	β	SE	*p*-Value	FDR-Adjusted *p*-Value	95% CI	β	SE	*p*-Value	FDR-Adjusted *p*-Value	95% CI
Premorbid IQ	+	−0.0305	0.01969	0.125	0.292	−0.06910, 0.00808	0.0096	0.01476	0.516	0.723	−0.01931, 0.03854
Language	+	−0.0034	0.00198	0.087	0.338	−0.00737, 0.00042	0.0017	0.00151	0.247	0.692	−0.00119, 0.00474
Executive Function	+	−0.0008	0.00142	0.541	0.757	−0.00366, 0.00191	0.0002	0.00106	0.805	0.846	−0.00183, 0.00236
Episodic Memory	+	−0.0007	0.00178	0.667	0.705	−0.00427, 0.00272	−0.0015	0.00132	0.258	0.596	−0.00409, 0.00107
Attention	+	−0.0028	0.00095	**0.004**	0.016 *	−0.00470, −0.00097	0.0012	0.00078	0.105	0.417	−0.00023, 0.00282
Recognition Memory	+	−0.0008	0.00185	0.639	0.829	−0.00451, 0.00276	−0.0005	0.00134	0.682	0.834	−0.00319, 0.00208
PACC	+	−0.0008	0.00077	0.284	0.521	−0.00236, 0.00068	−0.00008	0.00058	0.880	0.880	−0.00123, 0.00106
Global Cognition	+	−0.0005	0.00210	0.783	0.783	−0.00469, 0.00353	−0.0095	0.00154	0.538	0.628	−0.00398, −0.00207
Premorbid IQ	-	−0.0039	0.00874	0.649	0.998	−0.02112, 0.01316	−0.0019	0.00762	0.799	0.838	−0.01689, 0.01300
Language	-	0.0009	0.00085	0.267	0.623	−0.00072, 0.00262	0.0013	0.00077	0.088	0.309	−0.00018, 0.00286
Executive Function	-	0.0006	0.00054	0.257	0.450	−0.00044, 0.00167	0.0003	0.00051	0.558	0.781	−0.00070, 0.00131
Episodic Memory	-	−0.0006	0.00068	0.362	0.821	−0.00197, 0.00071	0.0003	0.00062	0.601	0.735	−0.00089, 0.00154
Attention	-	−0.0003	0.00050	0.452	0.568	−0.00137, 0.00060	0.0006	0.00046	0.163	0.308	−0.00026, 0.00157
Recognition Memory	-	−0.0010	0.00068	0.140	0.262	−0.00237, 0.00032	0.0004	0.00065	0.452	0.591	−0.00079, 0.00177
PACC	-	−0.00008	0.00028	0.750	0.750	−0.00064, 0.00046	−0.00004	0.00026	0.876	0.876	−0.00055, 0.00047
Global Cognition	-	−0.0005	0.00074	0.477	0.557	−0.00199, 0.00092	0.00001	0.00068	0.981	0.981	−0.00132, 0.00136

Abbreviations: *APOE*, apolipoprotein E; CI, confidence interval; FDR, false discovery rate; IQ, intelligence quotient; PACC, Preclinical Alzheimer’s Cognitive Composite; SE, standard error. Model includes age, sex, years of education (≤12 years or >12 years), energy intake and body mass index as independent variables. Bold indicates statistical significance (* FDR-adjusted *p* < 0.05). (≤12 years, or >12 years), energy intake and body mass index as independent variables.

## Data Availability

The data presented in this study are available from corresponding authors upon written, formal request. The data are held in a password-protected secure cloud environment based at our universities.

## Supplementary Materials

The following supporting information can be downloaded at: https://www.mdpi.com/article/10.3390/nu18162592/s1. Table S1: Descriptive statistics for the cohort based on stratification by those who did and did not develop cardiometabolic conditions; Table S2: Results of linear mixed effect model analyses examining the association between baseline diet scores and change in cognitive performance over 90 months excluding participants with baseline MoCA scores < 23. Standardised β values shown; Table S3: Results of linear mixed effect model analyses examining the association between baseline diet scores and change in cognitive performance over 90 months excluding participants with baseline MoCA scores < 23, stratified by APOE ε4 carrier status. Standardised β values shown; Table S4: STROBE Statement—Checklist of items that should be included in reports of cohort studies.

## Abbreviations

The following abbreviations are used in this manuscript:

AD	Alzheimer’s disease
APOE	Apolipoprotein E
APOE ε4	Apolipoprotein E epsilon 4 allele
BMI	Body Mass Index
CCVFFQ	Cancer Council of Victoria Food Frequency Questionnaire
CI	Confidence interval
CSF	Cerebrospinal fluid
CVLT-II	California Verbal Learning Test–Second Edition
D-KEFS	Delis–Kaplan Executive Function System
DNA	Deoxyribonucleic acid
EDTA	Ethylenediaminetetraacetic acid
FDR	False discovery rate
IQ	Intelligence Quotient
Kcal	Kilocalorie
MAR	Missing At Random
MoCA	Montreal Cognitive Assessment
PACC	Preclinical Alzheimer’s Cognitive Composite
PCA	Principal component analysis
PFC	Prefrontal cortex
RCFT	Rey Complex Figure Test
RCT	Randomized controlled trial
SE	Standard error
SPSS	Statistical Package for the Social Sciences
WAMS	Western Australia Memory Study
WHO	World Health Organization
Z-score	Standardized score
