# Supplementary material for: Dietary Patterns and Cognitive Decline in Older Adults: Findings from the Western Australia Memory Study
Source: Nutrients. 2026 Aug 7;18(16):2592. doi: 10.3390/nu18162592 (PMC13516335; doi:10.3390/nu18162592)
Supplement: Supplementary file 1 [file nutrients-18-02592-s001.zip › Supplementary Table S1.pdf]

Supplementary Table S1: Descriptive statistics for the cohort based on stratification by those who did and did not develop cardiometabolic conditions

	**Those who did not develop cardiometabolic conditions (n = 163)**	**Those who did develop cardiometabolic conditions (n = 22)**	***p*-values for differences in those who did and did not develop cardiometabolic conditions**
**Age, y**	70.5 ± 5.7	69.8 ± 6.5	.598
**Sex, men; n (%)**	111 (68.1)	15 (68.2)	.994
**Presence of *APOE* ε4 allele; n (%)**	50 (30.7)	7 (31.8)	.913
**Baseline body mass index[1], kg/m^2**	26.8 ± 4.4	26.7 ± 3.4	.945
**Baseline energy intake, Kcal**	1578.0 ± 532.3	1798.4 ± 554.0	.071
**Education ≤ 12 y; n (%)**	37 (22.7)	11 (50.0)	**.006**
**Baseline Western diet score**	185.9 ± 112.2	256.5 ± 195.3	**.013**
**Baseline Prudent diet score**	219.3 ± 98.0	245.8 ± 99.5	.236

If not otherwise described, data are presented as mean ± standard deviation of the mean.

Abbreviations: *APOE*, apolipoprotein E; Kcal, kilocalorie; Kg, kilogram; m^2, meter squared; y, years.

Bold indicates statistical significance ($p < .05$); Characteristics compared using independent samples t-test for continuous variables and χ^2 for categorical variables.

[1] Body Mass Index is calculated as weight in kilograms divided by height in meters squared.
