# Supplementary material for: Dietary Patterns and Cognitive Decline in Older Adults: Findings from the Western Australia Memory Study
Source: Nutrients. 2026 Aug 7;18(16):2592. doi: 10.3390/nu18162592 (PMC13516335; doi:10.3390/nu18162592)
Supplement: Supplementary file 1 [file nutrients-18-02592-s001.zip › Supplementary Table S2.pdf]

Supplementary Table S2. Results of linear mixed effect model analyses examining the association between baseline diet scores and change in cognitive performance over 90 months excluding participants with baseline MoCA scores <23. Standardised β values shown.

Cognitive domain	Western diet					Prudent diet				
	β	SE	*p*-value	FDR adjusted *p*-value	95% CI	β	SE	*p*-value	FDR adjusted *p*-value	95% CI
**Premorbid IQ**	0.0032	0.00818	.698	.957	-0.01286, 0.01922	-0.0014	0.00678	.837	.891	-0.01468, 0.01189
**Language**	0.0002	0.00079	.835	.841	-0.00139, 0.00172	0.0015	0.00070	.030	.224	0.00016, 0.00292
**Executive Function**	0.0003	0.00054	.613	.818	-0.00078, 0.00132	0.0004	0.00049	.390	.540	-0.00054, 0.00138
**Episodic Memory**	-0.0007	0.00064	.315	.503	-0.00191, 0.00061	-0.0001	0.00058	.866	.866	-0.00123, 0.00104
**Attention**	-0.0002	0.00042	.577	.659	-0.00107, 0.00059	0.0005	0.00039	.205	.515	-0.00027, 0.00125
**Recognition Memory**	-0.0010	0.00063	.116	.308	-0.00225, 0.00024	0.0004	0.00059	.524	.646	-0.00078, 0.00153
**PACC**	-0.0003	0.00037	.433	.692	-0.00101, 0.00043	0.0003	0.00033	.315	.420	-0.00031, 0.00098
**Global Cognition**	-0.0008	0.00062	.223	.356	-0.00199, 0.00046	0.0000	0.00060	.938	.938	-0.00112, 0.00121

Abbreviations: APOE, apolipoprotein E; CI, confidence interval; FDR, false discovery rate; IQ, intelligence quotient; MoCA, Montreal Cognitive Assessment; PACC, Preclinical Alzheimer's Cognitive Composite; SE, standard error.

Models were adjusted for age, sex, years of education (≤12 years or >12 years), energy intake and body mass index, total energy intake, and APOE genotype.
