# Supplementary material for: Dietary Patterns and Cognitive Decline in Older Adults: Findings from the Western Australia Memory Study
Source: Nutrients. 2026 Aug 7;18(16):2592. doi: 10.3390/nu18162592 (PMC13516335; doi:10.3390/nu18162592)
Supplement: Supplementary file 1 [file nutrients-18-02592-s001.zip › Supplementary Table S3.pdf]

Supplementary Table S3. Results of linear mixed effect model analyses examining the association between baseline diet scores and change in cognitive performance over 90 months excluding participants with baseline MoCA scores <23, stratified by APOE ε4 carrier status. Standardised β values shown.

Cognitive domain	APOE ε4 allele carrier status	Western diet					Prudent diet				
		β	SE	*p*-value	FDR adjusted *p*-value	95% CI	β	SE	*p*-value	FDR adjusted *p*-value	95% CI
**Premorbid IQ**	+	0.0016	0.02632	.951	.981	-0.04995, 0.05323	0.0043	0.02494	.864	.997	-0.04458, 0.05317
**Language**	+	-0.0035	0.00221	.125	.616	-0.00781, 0.00087	0.0019	0.00168	.255	.779	-0.00135, 0.00523
**Executive Function**	+	-0.0011	0.00156	.478	.660	-0.00417, 0.00194	0.0005	0.00117	.667	.778	-0.00179, 0.00280
**Episodic Memory**	+	-0.0012	0.00172	.479	.671	-0.00459, 0.00214	-0.0009	0.00128	.492	.689	-0.00341, 0.00162
**Attention**	+	-0.0017	0.00092	.066	.233	-0.00356, 0.00006	0.0010	0.00072	.196	.685	-0.00046, 0.00237
**Recognition Memory**	+	-0.0014	0.00185	.465	.965	-0.00500, 0.00227	-0.0005	0.00135	.706	.984	-0.00316, 0.00213
**PACC**	+	-0.0019	0.00131	.156	.758	-0.00448, 0.00065	0.0015	0.00111	.194	.852	-0.00069, 0.00365
**Global Cognition**	+	-0.0007	0.00336	.835	.994	-0.00729, 0.00588	-0.0005	0.00267	.856	.930	-0.00572, 0.00474
**Premorbid IQ**	−	0.0087	0.01010	.390	.455	-0.01108, 0.02852	-0.0110	0.00892	.221	.351	-0.02844, 0.00651
**Language**	−	0.0008	0.00085	.357	.824	-0.00088, 0.00247	0.0015	0.00077	.059	.208	-0.00004, 0.00298
**Executive Function**	−	0.0004	0.00059	.536	.751	-0.00079, 0.00153	0.0003	0.00055	.545	.763	-0.00075, 0.00142
**Episodic Memory**	−	-0.0007	0.00071	.302	.697	-0.00214, 0.00066	0.0004	0.00064	.570	.798	-0.00090, 0.00163
**Attention**	−	-0.0002	0.00051	.633	.800	-0.00124, 0.00076	0.0004	0.00047	.352	.822	-0.00048, 0.00136
**Recognition Memory**	−	-0.0012	0.00070	.089	.207	-0.00259, 0.00017	0.0006	0.00067	.361	.506	-0.00070, 0.00193
**PACC**	−	-0.0002	0.00046	.717	.779	-0.00106, 0.00073	0.0003	0.00049	.595	.694	-0.00070, 0.00122
**Global Cognition**	−	-0.0009	0.00085	.299	.523	-0.00254, 0.00077	-0.0002	0.00090	.814	.891	-0.00197, 0.00155

Abbreviations: APOE, apolipoprotein E; CI, confidence interval; FDR, false discovery rate; IQ, intelligence quotient; MoCA, Montreal Cognitive Assessment; PACC, Preclinical Alzheimer’s Cognitive Composite; SE, standard error.

Models were adjusted for age, sex, years of education (≤12 years or >12 years), energy intake and body mass index, and total energy intake.

APOE ε4 carrier status: +, carrier; −, non-carrier.
